# Monitoring of some pesticides residue in consumed tea in Tehran market

**DOI:** 10.1186/1735-2746-10-9

**Published:** 2013-01-12

**Authors:** Maryam Amirahmadi, Shahram Shoeibi, Mehdi Abdollahi, Hossein Rastegar, Roya Khosrokhavar, Morteza Pirali Hamedani

**Affiliations:** 1 Food and Drug Laboratory Research Center, Food and Drug Organization, MOH & ME, Tehran, Iran; 2 Food and Drug Reference Control Laboratories Center, Food and Drug Organization, MOH & ME, Tehran, Iran; 3 School of Pharmacy, Tehran University of Medical Sciences, Tehran, Iran; 4 Department of Pharmacology, School of Pharmacy, Tehran University of Medical Sciences, Tehran, Iran

**Keywords:** Pesticides residue, Tea, GC/MS, Spiked calibration curve

## Abstract

Tea is an agricultural product of the leaves, leaf buds, and internodes of various cultivars and sub-varieties of the Camellia sinensis plant, processed and vulcanized using various methods. Tea is a main beverage in Iranian food basket so should be free from toxic elements such as pesticides residue. There is no data bank on the residue of pesticides in the consumed black tea in Iran. The present study is the first attempt for monitoring of 25 pesticide residues from different chemical groups in tea samples obtained from local markets in Tehran, I.R. Iran during the period 2011. A reliable and accurate method based on spiked calibration curve and QuEChERS sample preparation was developed for determination of pesticide residues in tea by gas chromatography–mass spectrometry (GC/MS). The using of spiked calibration standards for constructing the calibration curve substantially reduced adverse matrix-related effects and negative recovery affected by GCB on pesticides. The recovery of pesticides at 3 concentration levels (n = 3) was in range of 81.4 - 99.4%. The method was proved to be repeatable with RSDr lower than 20%. The limits of quantification for all pesticides were ≤20 ng/g. 53 samples from 17 imported and manufactured brand were analyzed. Detectable pesticides residues were found in 28.3% (15 samples) of the samples. All of the positive samples were contaminated with unregulated pesticides (Endosulfan Sulfate or Bifenthrin) which are established by ISIRI. None of the samples had contamination higher than maximum residue limit set by EU and India.

## Introduction

In recent years, a body of scientific evidence has shown that regular tea drinking may have an important role in health and wellness [1]."Tea" refers to the aromatic beverage prepared from the cured leaves by combination with hot or boiling water [2]. Tea contains catechins, a type of antioxidant [3], theanine and the stimulant caffeine at about 3% of its dry weight [4]. Tea has negligible carbohydrates, fat, and protein. Presence of different alkaloids, pigments, lipids and proteins cause difficulties in pesticides analysis in tea [5]. Tea also called as ‘health beverage’ because of its antioxidant properties and beneficial effects on human health so, should be free from contaminants such as pesticides residue [6]. Most tea farmers and producers may use pesticides for agricultural products protection [5].

In pesticides analysis some strategies have been applied in method development of tea analysis, and recently there have been many crops pesticide residues analysis methods were developed for use on tea, especially using GC and LC instruments [7-17].

The presence of pigments in tea cause problems in purification of extract which lead to use some absorbents such as Graphite Carbon Black (GCB) for purification. GCB, however, negatively affected the recovery of planar pesticides. To solve this problem, toluene was added during cleanup as noted by Zhao and Stevens [18]. Other than residues the extracts contain many other components, such as pigments, lipids, and alkaloids, which can severely interfere with pesticides analysis [5].

Tea is one of the important imported commodities in Iran and is a main beverage in Iranian food basket especially breakfast. Currently, consumption of tea as a soft drink is very popular in Iran and its production in 2006 was about 78,000 tons [19]. The use of pesticides on this crop in exported country had largely been guided by the MRL or the tolerance limits prescribed by their national authority, the European Union (EU) or Codex Alimentarius Commission of FAO/WHO. However, the national laws of tea exporting countries vary on the MRL of different pesticides on tea. In the other words, there is no harmonization of the MRL for pesticides in different area of tea cultivated. It may be caused difference climate and so different pests exist in each area.

In this work we have studied presence of pesticides in one of the most consumed beverage, tea, from food safety aspect. In Iran there are MRLs for three pesticides in tea but there is no data bank on the residues of pesticides in consumed black tea. Therefore, this study is a survey which is conducted to assess the status of pesticides residue in produced and imported tea with in the different brands in Tehran market during 2011.

The selected pesticides are twenty-five GC-amenable which are presented in Table 1. In this paper we used a rapid multi-residue method of analysis based on a QuEChERS procedure using toluene and ethanol (50:50, v/v) for extraction, and PSA and GCB for purification and using spiked calibration curve to simultaneously determine pesticides residues in tea. 

**Table 1:** The retention time, diagnostic ions and selected quantification ion for the target pesticides and internal standard

**Compound**	**Diagnostic ions**	**Quantification ion**	**Retention time (min)**
Carbaryl	115.1, 144.1, 116.1	144.1	19.75
Gamma HCH	221, 219, 217	219	25.89
Diazinon	304.1, 276, 227.1	304.1	26.11
Pirimicarb	238, 166, 167	166	27.25
Chlorpyrifos-methyl	290, 288, 286	286	28.37
Pirimiphos-methyl	305.1, 290.1, 276.1	290.1	29.32
Fenitrothion	277.1, 278.1, 260	277.1	29.41
Malathion	173.1, 158, 124.9	173	29.65
TPM*	244.1, 158, 124.9	244.1	30.74
Procymidone	284, 285, 283	283	32.31
Alpha-Endosulfan	338.9, 264.9, 240.9	240.9	33.43
Iprodione	244, 187, 246	187	35.51
Beta-Endosulfan	338.9, 266.9, 240.9	240.9	35.62
Propiconazole 1	261, 259, 173	259	36.80
Propiconazole 2	261, 259, 173	259	37.01
Endosulfan sulfate	386.9, 273.8, 271.8	271.8	37.19
Bifenthrin	181.2, 182.1, 165.1	181.2	38.61
Lambda cyhalothrin	208, 197, 181	181	40.51
Permethrin 1	183.1, 184.1, 163	183.1	42.32
Permethrin 2	183.1, 184.1, 163	183.1	42.59
Cypermethrin1	207, 208.1, 281	207	44.57
Cypermethrin 2	207, 208.1, 281	207	44.80
Cypermethrin 3	207, 208.1, 281	207	45.11
Difenoconazole 1	265, 267, 323	323	49.44
Difenoconazole 2	265, 267, 323	323	49.73
Deltamethrin	252.9, 254.9, 209.1	252.9	50.98

* Triphenylmethane as internal standard.

## Materials and methods

### GC–SQ/MS

An Agilent Technologies 6890 N Network GC System chromatograph (Wilmington, USA) with a SQ detector and equipped with an Agilent 7683B autosampler (Agilent technologies, USA) and a HP-5 capillary column (30 m × 0.25 mm I.D., 1 μm film thickness) were used for separation.

### Chemicals

All organic solvents, intended for extraction, were at least LC grade and purchased from Merck (Darmstadt, Germany). Primary Secondary Amine (PSA) and Graphite Carbon Black (GCB) were purchased from Supelco (Bellefonte, USA). Bulk quantities of NaCl were obtained from Merck (Darmstadt, Germany). All pesticide standards were purchased from Dr. Ehrenstorfer Co. (Augsburg, Germany). Anhydrous MgSO_4_ was obtained from SIGMA-Aldrich CO. (Japan). The MgSO_4_ was baked for 5 h at 500°C in a furnace to remove phthalates and residual water.

### Calibration

Individual stock standard solutions (1 mg/ml) were prepared in ethyl acetate and stored in the dark at −20°C. A mixed stock standard solution of pesticides was prepared in ethyl acetate at 10 μg/ml with respect to each pesticide. Spiked calibration curves at 7 levels of 10, 25, 50, 100, 200, 300 and 500 ng/g triplicate were prepared by addition of 5 μl, 12.5 μl, 25 μl, 50 μl, 100 μl, 150 μl and 250 μl of mixed standard stock solution, respectively, to 5 g of blank tea samples in each case.

A stock solution of triphenylmethane (TPM) in ethyl acetate at concentration of 1 mg/ml was used as internal standard and an aliquot of 10 μl of TPM solution in ethyl acetate was added to the spiked tea sample. The samples then were treated as described in next section.

### Sample preparation

5 g of milled black tea sample was weighted in a 50 ml falcon tube and 12.5 ml toluene and ethanol (50:50) was added for extraction. High speed vortex mixer was applied for mixing along 1 min. 0.5 g of NaCl was added to the mixture, and mixing was continued for an additional 1 min. The mixture was centrifuged for 5 min at 4000 rpm at −5°C. The supernatant was transferred to a 15 ml falcon tube containing 0.75 g MgSO_4_, 200 mg PSA and 200 mg GCB. After shaking for 1.5 min and centrifugation for 5 min at 4000 rpm at −5°C, 4 ml of supernatant was transferred to a 5 ml vial and evaporated to dryness under a gentle stream of nitrogen gas. The residue was reconstituted by toluene to obtain 1 ml solution, and after shaking for 3 min, 2 μl of the solution was injected into gas chromatograph.

### Recovery studies

For recovery determination, spiked blank tea samples at concentration levels of 30 ng/g, 60 ng/g and 250 ng/g were prepared in triplicates and kept for 1 h at ambient temperature prior to their use and then treated according to the procedure described in sample preparation section. The recoveries were calculated using the calibration curves obtained from spiked samples.

### GC-SQ–MS analysis

The GC-SQ-MS was applied with helium as carrier gas at a constant flow of 1 ml/min. The oven temperature started at 75°C and remained at this temperature for 3 min increasing to 120°C at 25°C/min ramp rate and then increased to 300°C at 5°C/min ramp, holding at 300°C for 11 min. Injection port was adjusted at 250°C and splitless mode was used.

After acquisition of the total ion chromatogram for the mixed stock standard solutions in scan mode, peaks were identified by their retention time and mass spectra. The most abundant ion that showed no evidence of chromatographic interference and had the highest signal-to-noise ratio was selected for quantification purposes.

### Quantitation

The concentrations of pesticides were determined by interpolation of the relative peak areas for each pesticide to internal standard peak area in the sample on the spiked calibration curve.

### Analysis of *real samples*

Fifty three black tea samples from 17 various brands were collected from the main market places in Tehran in 2011. The samples were randomly selected according to brands (imported or manufactured) which exist in market. In order to avoid any possible thermal decomposition of pesticide residues, 200 g tea sample was mixed with 100 g dry ice and milled with Romer mill (Stylemaster Drive, USA). 5 g of sample was subjected to the process of sample preparation (n = 1) which is described in sample preparation section.

## Results

### Gas chromatographic determination

**Figure 1:**
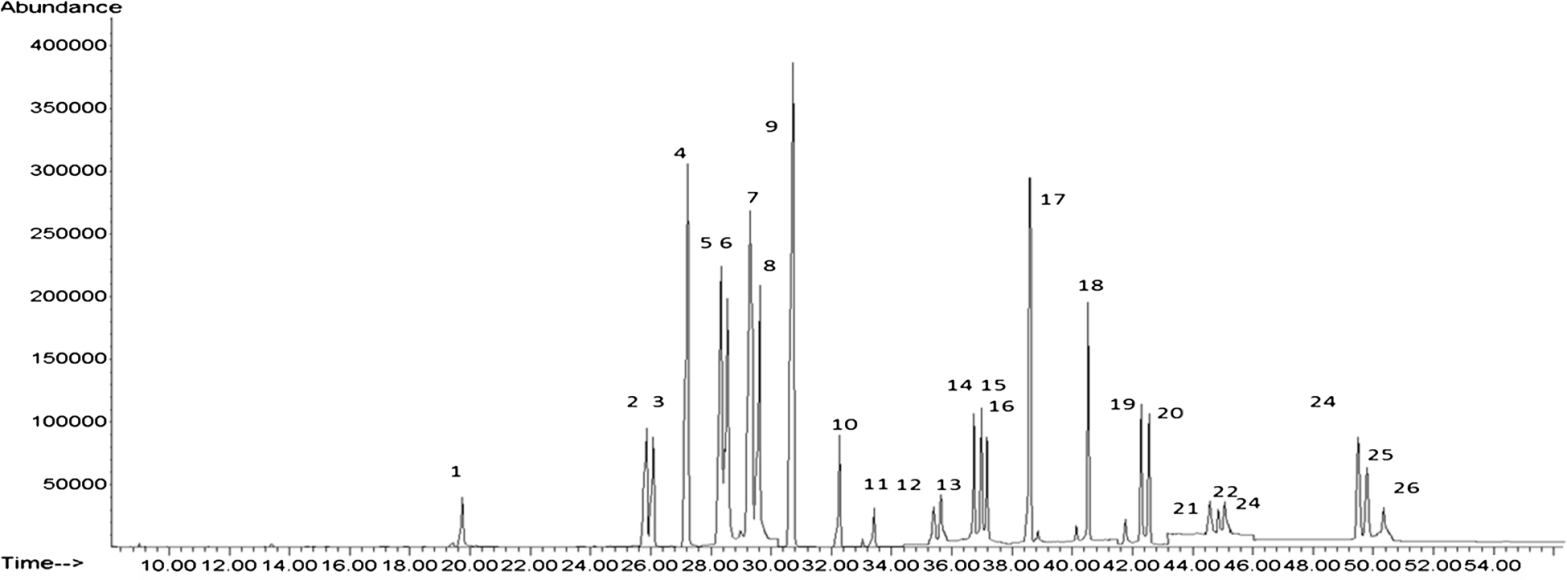
A representative chromatogram obtained for the 25 pesticides and internal standard (TPM), 1) Carbaryl; 2) Gamma HCH; 3) Diazinon; 4) Pirimicarb; 5) Chlorpryfos-methyl;6) Pirimiphos methyl; 7) Fenitrothion; 8) Malathion; 9) TPM(Istd); 10) Procymidon; 11) α -Endosulfan; 12) Iprodion; 13) β-Endosulfan; 14)Propiconazole1; 15) Propiconazole 2; 16) Endosulfan sulfate; 17) Bifenthrin; 18)L-cyhalothrin; 19) Permethrin 1; 20) Permethrin 2; 21) Cypermethrin 1; 22) Cypermethrin 2; 23) Cypermethrin 3; 24) Difenoconazole 1; 25) Difenoconazole 2 and 26) Deltamethrinin spiked tea at 200 ng/g in SIM mode.

Analysis was performed in the SIM mode based on the use of one target and two-three qualifier ions. Pesticides were identified according to their retention times and target and qualifier ions. The quantitation was based on the peak area ratio of the targets to that of internal standard. Table 1 summarizes studied pesticides with their diagnostic and qualifier ions used in SIM mode. GC–SQ–MS chromatogram of 25 pesticides and internal standard (TPM) analyzed in spiked tea is shown in Figure 1. 

### Method validation

#### Linearity of the calibration curves

All pesticides showed linearity in SIM mode. Linear spiked calibration curves for all the interest pesticides were obtained with correlation factors >0.997.

### Limits of detection and limits of quantification

Limits of detection (LODs) and limits of quantification (LOQs) of the proposed method were measured in spiked samples and calculated by considering a value 3 and 10 times of background noise, which for all pesticides were ≤5 ng/g and ≤20 ng/g, respectively.

#### Recovery

Table 2 presents the recovery and repeatability for three concentration levels of pesticides. The recovery of pesticides at 3 concentration levels triplicates was in the range of 81.4 - 99.4%. In terms of repeatability, the majority of the pesticides gave RSD < 20%. The recoveries and repeatabilities are in accordance with the criteria set by SANCO Guideline [20]. 

**Table 2:** Average recoveries (%) and range of relative standard deviations (%) of pesticides obtained by GC- MS analysis of Tea samples at 3 spiking levels (n = 3)

**Pesticide**	**Average recovery (%) (n = 3)**			**Total recovery (%) (n = 9)**	**Range 0f RSDr (%)**
	**30 ng/g**	**60 ng/g**	**250 ng/g**		
Carbaryl	81.2	79.9	83.2	81.4	5.6- 12.5
Gamma HCH	85.3	89.7	93.7	89.5	3.2-5.9
Diazinon	89.2	92.3	106.9	96.1	5.1-11.5
Pirimicarb	79.5	101.3	111.6	97.4	4.5- 8.9
Chlorpyrifos-methyl	88.7	94.3	89.5	90.8	0.6- 4.5
Fenitrothion	97.3	88.7	109.6	98.5	0.7-5.3
Pirimiphos-methyl	91.3	89.9	105.7	95.6	1.1-6.8
Malathion	86.8	93.6	108.9	96.4	2.3- 8.2
Alpha-Endosulfan	91.2	95.7	111.3	99.4	4.2-9.9
Procymidone	79.8	85.6	83.3	82.9	1.5-6.7
Iprodione	78.3	76.4	89.7	81.4	3.4-11.6
Beta-Endosulfan	91.3	89.4	105.4	95.4	3.4- 7.8
Endosulfan sulfate	91.3	89.6	98.6	93.2	3.8-9.7
Bifenthrin	78.6	88.3	95.6	87.5	1.8- 5.6
Propiconazole	101.2	98.7	95.9	98.6	0.9-3.4
Lambda cyhalothrin	92.1	98.2	101.6	97.3	1.5-6.5
Permethrin	95.7	89.9	112.1	99.2	3.4- 7.5
Cypermethrin	78.3	81.3	85.9	81.8	6.7-12.8
Difenoconazole	96.7	88.9	101.3	95.6	4.3-10.2
Deltamethrin	76.8	79.3	115.6	90.6	6.6- 15.6

#### Pesticides residue in real samples

Fifty three samples were milled and analyzed according to the above described method. Fifteen (28.3%) of the 53 samples showed contamination with pesticides (Table 3). 

**Table 3:** Pesticides residue and their concentrations in domestic and imported tea samples in Tehran, Iran

**Sample**	**Source of Tea**	**Pesticide**	**Concentration (μg/g)**	**LOQ (μg/g)**	**MRL (μg/g)**
1	Domestic	Endosulfan sulfate	0.007	0.005	5
2	Imported	Endosulfan sulfate	0.015	0.005	5
3	Domestic	Endosulfan sulfate	0.012	0.005	5
4	Domestic	Endosulfan sulfate	0.020	0.005	5
5	Imported	Endosulfan sulfate	0.010	0.005	5
6	Imported	Endosulfan sulfate	LOQ<	0.005	5
7	Domestic	Endosulfan sulfate	LOQ<	0.005	5
8	Imported	Endosulfan sulfate	0.007	0.005	5
9	Imported	Endosulfan sulfate	0.008	0.005	5
10	Imported	Endosulfan sulfate	LOQ<	0.005	5
11	Domestic	Bifenthrin	0.035	0.01	5
12	Domestic	Bifenthrin	LOQ<	0.01	5
13	Domestic	Bifenthrin	0.020	0.01	5
14	Imported	Bifenthrin	0.017	0.01	5
15	Domestic	Bifenthrin	0.019	0.01	5

Table 3 shows, none of the samples had contaminated with more than one pesticide and ten of the samples had contamination with endosulfan sulfate and five samples with bifenthrin. The concentrations of endosulfan sulfate and bifenthrin were below the MRLs of these pesticides in tea in EU and India. There is no MRL for the detected pesticides in tea in Iran.

## Discussion

Tea is cultivated in about 36 countries all over the world, but production is heavily concentrated in just a handful. In 2000, five countries (India, China, Sri Lanka, Kenya and Indonesia) together produced almost 80% of the world’s tea [21].

Some contaminants are important in tea, from food safety aspects, such as heavy metals and pesticides.

The unsafe use of chemicals not only puts the workers and the environment in danger, but also leaves traces of harmful pesticides and insecticides in the processed tea. According to a report published in the Economic Times (January 4, 1999), the European Tea Committee in its findings claimed a high incidence of pesticides in Indian tea exported to overseas markets [22].

There are some methods and strategies for control and monitoring related pesticides which used in tea.

Some studies focused on evaluation of a variety of solvents for extraction, such as acetonitrile, methanol, isopropanol, and multiple d-SPE cleanup methods, including C18, PSA, GCB, MgSO4, and their combinations for improvement of pesticide analysis method in tea. These conditions product extracts from clear solutions to dark-colored and cloudy suspensions [5]. GCB is used to remove pigments; GCB can also have a cause negative effect in the recovery of planar pesticides. To improvement this problem, toluene was added as extraction solvents [18].

In the present study, we used spiked calibration curve approach to overcome on both matrix and GCB negative effect. In this approach, calibration curves are prepared by the addition of standard solution to blank tea samples and these samples subjected to the same sample preparation procedure which is intended to be used for unknown samples. The results of reproducibility of recovery, as indicated by standard deviations, show that the applied method achieves excellent quality and reliability of results for a wide range of pesticides in accordance with the criteria set by SANCO Guideline could be considered reliable enough for the analysis of tea [20].

Results of the survey had shown that consumed tea in Iran are rather free from residues of pesticides or so lower than established MRLs which presented that pesticides may not used or the currence period is spend. Analysis of 53 samples in 2011 showed the presence of endosulfan sulfate in 18.9% of the samples with residues ranged from 5–20 μg/kg and the presence of bifenthrin in 9.4% of the samples with residues ranged from 5–35 μg/kg, these contaminated samples were lower than MRL established by regulation authorities from India and England. None of the samples had residues of other interest pesticides (Table 3). In a study by Subbiah Seenivasan and NarayananNair Muraleedharan in 2011 a large scale survey of produced tea in the factories of south India had been carried out for a period of three years from 2006 to 2008 and 912 samples were analysed for the residues of certain pesticides such as dicofol, ethion, quinalphos, hexaconazole, fenpropathrin, fenvalerate and propargite. The analytical data showed that only less than 0.5% of samples had residues of interested pesticides. Bishnu etal, 2009 quantified the residues of 7 pesticides in tea ecosystems of Dooars regions of West Bengal, India. They found that the residues of banned pesticides like heptachlor in tasted samples may pose health hazard to the consumer [23]. All the regulatory authorities have established the maximum residue levels (MRL) of pesticides in black/green tea since these are the commodities being traded [6].

In our present survey we found the residues of endosulfan sulfate and bifenthrin were found in 28.3% of tea samples, though none of sample it does exceed the MRL set by EU and India (Table 3).

## Conclusion

An accurate, precision and reliable method was developed to determine 25 pesticides residue in tea; a main drink in Iranian food basket. The method which consists of a QuEChERS sample preparation and GC-SQ-MS-SIM analysis showed a high sensitivity and confirmatory power necessary for the determination of pesticides residue at low levels. The excellent results of method validation show the applied strategy for determination and quantification of pesticides residue in tea and other matrices is reliable, useful and acceptable. Contamination of 23.8% of the analyzed tea samples with unregulated pesticides calls for the routine monitoring programs for analysis of pesticide residues in tea and review for MRL by regulatory bodies in IRAN. According to our results, the detected pesticides are not in the list of Iranian authorities [24] which it may be caused that most of the consumed tea in Iran is imported and also most of the domestic tea which are packed in manufactures, use from imported bulk tea, so it should be considered the regulation of the other countries which export tea to Iran. The geographic properties of each country may identify the pests, so the used pesticides are very different and control of safety from this aspect should be necessary. Also the lists of pesticides and their MRLs, according to the found data, national and international regulations should be periodically revised.

## Competing interest

The authors declare that they have no competing interests.

## Authors’ contributions

**MA** is contributed in set up and validation the method, Instrumental analysis, Preparation of standard solutions drafting the manuscript. **Sh Sh**, planning and programming the proposal, food and toxicology consultation, editing the manuscript. **MA**, sample preparation, sample collection Instrumental analysis, Preparation of standard solutions. **HR**, Main supervisor, head of scientific team. **R Kh**, Food and Toxicology consultant. Statistical analysis of collected data. **MP**, Main supervisor, Planning and programming the proposal. All authors read and approved the final manuscript.
